# PDE4 Inhibitors: Profiling Hits through the Multitude of Structural Classes

**DOI:** 10.3390/ijms241411518

**Published:** 2023-07-15

**Authors:** Jian Jin, Francesca Mazzacuva, Letizia Crocetti, Maria Paola Giovannoni, Agostino Cilibrizzi

**Affiliations:** 1 Institute of Pharmaceutical Science, King’s College London, Stamford Street, London SE1 9NH, UK; jian.jin@kcl.ac.uk; 2 School of Health, Sport and Bioscience, University of East London, London E15 4LZ, UK; f.mazzacuva@uel.ac.uk; 3 Neurofarba Department, Pharmaceutical and Nutraceutical Section, Via Ugo Schiff 6, Sesto Fiorentino, University of Florence, 50019 Florence, Italy; letizia.crocetti@unifi.it (L.C.); mariapaola.giovannoni@unifi.it (M.P.G.); 4 Centre for Therapeutic Innovation, University of Bath, Bath BA2 7AY, UK

**Keywords:** phosphodiesterases 4, PDE4 inhibitors, structural analysis, PDE4 selectivity, respiratory diseases, dual activity

## Abstract

Cyclic nucleotide phosphodiesterases 4 (PDE4) are a family of enzymes which specifically promote the hydrolysis and degradation of cAMP. The inhibition of PDE4 enzymes has been widely investigated as a possible alternative strategy for the treatment of a variety of respiratory diseases, including chronic obstructive pulmonary disease and asthma, as well as psoriasis and other autoimmune disorders. In this context, the identification of new molecules as PDE4 inhibitors continues to be an active field of investigation within drug discovery. This review summarizes the medicinal chemistry journey in the design and development of effective PDE4 inhibitors, analyzed through chemical classes and taking into consideration structural aspects and binding properties, as well as inhibitory efficacy, PDE4 selectivity and the potential as therapeutic agents.

## 1. Introduction

Cyclic 3′,5′-adenosine monophosphate (cAMP) and cyclic 3′,5′-guanosine monophosphate (cGMP) are two second messenger molecules that are involved in transducing the action of neurotransmitters, hormones and other cellular effectors, thus contributing to the regulation of various physiological processes [1,2,3]. These nucleotides can also amplify the signal strength of many functional responses by binding to intracellular regulatory targets [3,4,5,6,7]. Several studies have shown that the low intracellular level of cAMP has a role in the pathogenesis of respiratory disorders, such as chronic obstructive pulmonary disease (COPD) and asthma, as well as diseases characterized by a dysregulation of the immune system, including psoriasis and other chronic inflammatory conditions. Consequently, new therapeutic strategies focusing on the increase of cellular cAMP levels have attracted extensive medical interest in recent times [8,9,10], to improve current protocols for the treatment of these diseases.

Cyclic nucleotide phosphodiesterases (PDEs) are a family of enzymes which operate the hydrolysis and degradation of cAMP and cGMP. Based on their structure, sequence homology and selectivity for cAMP or cGMP, PDEs are classified into 11 distinct families. Within these, phosphodiesterases 4 (PDE4) consist of four subfamilies (PDE4A–PDE4D), which, in general, are predominantly expressed in brain, immune system cells and cardiovascular tissues (although significantly varied distribution and regulatory properties have also been reported for them) [11,12,13]. In turn, each PDE4 subfamily includes a number of subtypes encoded by unique genes. For instance, the PDE4B subfamily comprises five isoforms (PDE4B1-5) [14], each having different levels of expression, as well as functions, in cells and tissues [15]. By catalyzing the hydrolysis of cAMP, PDE4 have a crucial role in interfering with the release and action of pro- and/or anti-inflammatory mediators, such as tumor necrosis factor α (TNF-α), interleukin 10 (IL-10) and interleukin 12 (IL-12) [15], with several implications in regulating inflammatory responses [16,17,18,19]. In this regard, PDE4A, PDE4B, and PDE4D have been found highly expressed in the majority of immune system cells, where PDE4C are largely absent [20]. Moreover, PDE4B and PDE4D are involved in the modulation of neutrophil function, thus attracting considerable interest as pharmacological targets in drug discovery programs focusing on the identification of novel inhibitors as anti-inflammatory drugs [21,22,23].

**Figure 1:**
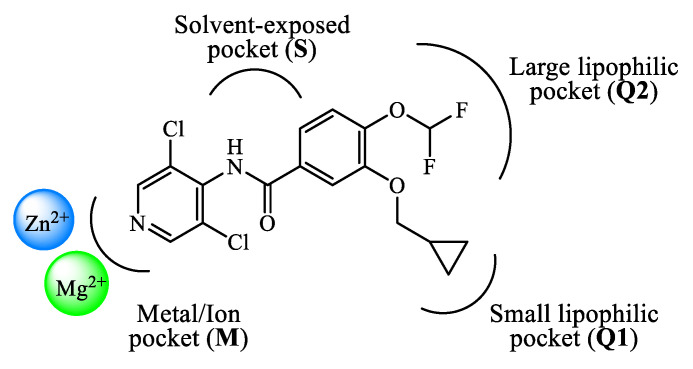
Schematic representation of the interactions of roflumilast in the PDE4 active site.

The design and development of PDE4 inhibitors as drug candidates dates back to the 1980s [24,25]. Successful agents demonstrated to specifically interact with the PDE4 active site [26], which has been established to consist of a divalent metal pocket (i.e., ‘M pocket’, for Zn^2+^ and Mg^2+^ that operate the hydrolysis of cAMP), two Q pockets (i.e., ‘Q1 and Q2 pockets’, characterized by the presence of hydrophobic residues) and a solvated pocket (i.e., ‘S pocket’, a solvent filled region characterized by the presence of polar residues). More in detail, further structural analysis highlighted that: (i) the M pocket contains highly conserved hydrophobic and polar residues which coordinate the metal ions; (ii) the Q1 pocket is a small hydrophobic pocket pointing away from the S pocket, while the Q2 pocket results larger than Q1 and adjacent to the S pocket; (iii) the S pocket consists mainly of hydrophilic amino acids and is filled with a network of water molecules which have a role in the formation of enzyme-inhibitor complexes in several cases. These four pockets can be occupied by the inhibitors through a series of binding interactions (as an, e.g., Figure 1 depicts the interactions of roflumilast, a first generation PDE4 inhibitor, in the active site of the enzyme), for instance hydrophobic interactions with conserved phenylalanine and isoleucine residues have been widely reported [27]. Furthermore, several inhibitors have been shown to occupy the active site in the region surrounding the Q pockets by establishing three main interactions: (i) coordination to the metal ions, often mediated by molecules of water; (ii) hydrogen bonds within the ‘nucleotide recognition area’; (iii) interactions with various hydrophobic residues in different parts of the active site. Overall, the therapeutic properties of PDE4 inhibitors are linked to their ability to suppress the activity of various endogenous mediators of inflammation (e.g., TNF-α, IL-10, and IL-12), as well as to inhibit the expression and elevation of cell adhesion molecules participating to the immune response [28], and the possible clinical value of many of them has been assessed in a variety of trials for several disease conditions.

**Figure 2:**
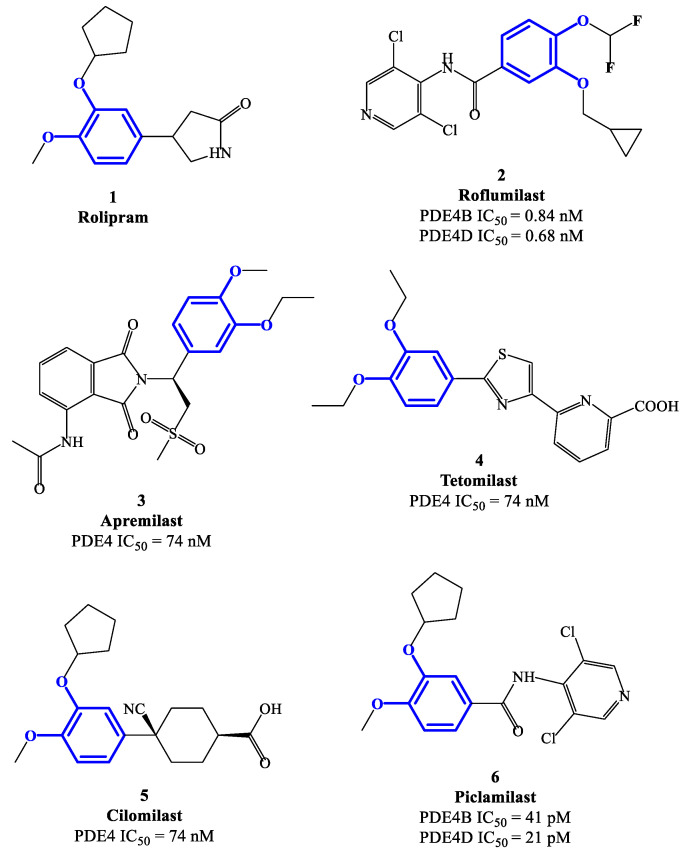
Chemical structures of PDE4 inhibitors based on a catechol-ether scaffold.

From the 1980s, the development of new PDE4 inhibitors has progressed at a breakneck pace, including the following milestone discoveries. Theophylline was one of the early discovered agents having weak activity and no selectivity towards the different PDE subfamilies (as well as antagonist effects on A1 and A3 adenosine receptors), initially used in the clinics to treat asthma and COPD. At present time, theophylline is no longer a first line agent in the management of these conditions, but its use is limited to cases where other bronchodilator agents fail to respond to medical treatment [29]. Rolipram 1 (Figure 2) is a selective PDE4 inhibitor also developed in the early phases (i.e., 1990s) and extensively studied for its anti-inflammatory action, in particular for the treatment of asthma [24,30]. Its clinical use has been hampered due to the severe side effects, mostly affecting the gastrointestinal and central nervous systems [31]. Rolipram is currently used as a prototype reference compound in pharmacology studies to evaluate the activity of new PDE4 inhibitors. Roflumilast 2 (Figure 1 and Figure 2), a second-generation PDE4 inhibitor, has been approved in the EU (in 2010) for the treatment of COPD, as well as in USA (in 2022) to treat plaque psoriasis [32,33,34]. Roflumilast has high inhibitory activity and selectively towards PDE4 isoenzymes, with no effects on the other PDE families. After roflumilast, three other PDE4 inhibitors have been approved for clinical use, namely apremilast (Figure 2) for psoriatic arthritis, crisaborole for atopic dermatitis and ibudilast for Krabbe disease [35,36,37,38,39]. In this journey, many clinical trials based on PDE4 inhibitors as potential new drugs have been conducted and then terminated due to narrow therapeutic windows in combination with substantial side effects. This is mainly determined by the poor selectivity of several agents towards PDE/PDE4 subfamilies and subtypes, as well as a low specificity in terms mechanism of action in general [40,41]. Thus, the development of novel and specific PDE4 inhibitors remains an important field of investigation to identify more effective drug candidates that can be translated into modern anti-inflammatory and/or immunoregulatory pharmaceuticals. This review focuses on a selection of relevant medicinal chemistry steps in the development of PDE4 inhibitors (including dual agents, gaining wide interest in the field in recent times), which are analyzed through the various chemical classes and evaluated in terms of structural and pharmacophore features, binding properties, inhibitory activity, as well as potential therapeutic applications.

## 2. PDE4 Inhibitors

### 2.1. Catechol-Ether Derivatives

Rolipram **1** and roflumilast **2** (Figure 2), introduced above, are two PDE4 inhibitors characterized by a catechol-ether moiety. Rolipram has been demonstrated to interact with a conserved glutamine across the PDE families, forming a hydrogen bond that inhibits the binding between the enzymes and the adenine residue in cAMP [42]. Moreover, it contains a methoxy group and a cyclopentyloxy group which can establish additional interactions, by occupying the hydrophobic pockets Q1 and Q2, respectively [43]. Roflumilast **2**, a highly selective PDE4 inhibitor, was the first PDE4 inhibitor approved for medical use in the treatment of severe COPD. In earlier studies, it was demonstrated that roflumilast had highly positive effects in the treatment of respiratory diseases, for instance by suppressing lung inflammation and leukocyte infiltration, as well as protecting the airways by stimulating the mucociliary clearance [40]. As a result, this agent was further evaluated in several clinical trials for various diseases, including asthma, bronchial asthma and noncystic fibrosis bronchiectasis, demonstrating also a lower incidence of possible adverse effects in comparison to early developed PDE4 inhibitors [40]. Between the PDE4 subtypes, roflumilast mainly inhibits PDE4B and PDE4D, with IC_50_ = 0.84 and 0.68 nM, respectively [27], while higher concentrations (i.e., in the μM range) are required to detemine inhibitory effects on PDE4A and PDE4C [40,44,45], and this may have an impact on the more favorable profile as a drug. Structurally, cyclopropylmethoxy and difluoromethoxy groups are present on the catechol-ether function, which are reported to interact with the hydrophobic Q_1_ and Q_2_ pockets in the PDE4 active site (Figure 1), in a similar fashion to rolipram. Several PDE4 inhibitors have been developed on the basis of structural modifications of roflumilast, for instance replacing the methyl cyclopropane with other groups, or modifying the amide linker between the dichloropyridine and catechol residue, although they were generally not suitable for medical use [27].

Apremilast **3** (Figure 2) is an oral PDE4 inhibitor with IC_50_ = 74 nM, which was approved by FDA in 2014 and mainly used for the treatment of psoriasis and other chronic immune-mediated diseases [46]. Differently to other inhibitors of this class, apremilast contains in its structure a phthalimide ring which may participate in the formation of additional hydrophobic interactions in the PDE4 active site, as well as contribute to the high selectivity towards PDE4 [47]. Additionally, an ethoxy group is introduced in position 3 of the catechol-diether moiety in apremilast, replacing the cyclopentyloxy residue of rolipram which was considered the main determinant of the binding to an accessory site in PDE4 [48,49], namely the so-called ‘rolipram binding site’. High-affinity for this additional site was reported to produce both enhanced inhibitory potency and, unfavorably, undesired adverse reactions (e.g., nausea, vomiting and increased gastric acid secretion) [50]. This structural change opens possibilities of exploring additional modifications on apremilast, having the potential to generate more effective, as well as safer, PDE4 inhibitors.

Tetomilast **4** (Figure 2) is a thiazole-based PDE4 inhibitor with IC_50_ = 74 nM, which has been reported to stimulate the expression of anti-inflammatory mediators, such as IL-10 and prostaglandin E2, and inhibit the release of TNF-α and IL-12. Moreover, this drug was originally investigated for the treatment of COPD and inflammatory bowel disease, but Phase II and III clincal trials [51] were not followed by FDA approval due to the lack of clear evidence in terms of modification of the disease progression. Cilomilast **5** (Figure 2) is a second-generation PDE4 inhibitor exhibiting anti-inflammatory activity, along with favorable effects during bronchoconstriction, mucus hypersecretion and airway remodeling associated with COPD (whereby an extensive clinical evaluation has been conducted in the 2000s) [24,35]. Piclamilast **6** (Figure 2) was developed through structural hybridization of rolipram **1** and roflumilast **2**, resulting in very high inhibitory activity on PDE4B and PDE4D, namely IC_50_ = 41 and 21 pM, respectively [36]. Structurally, the methoxy and cyclopentyloxy groups are reported to occupy the two Q pockets in the active site of the enzyme (in agreement with the binding mode of rolipram), while the dichloropyridyl group (derived from the structure of roflumilast) resulted in the formation of a hydrogen bond in the metal pocket [38]. However, the clinical translation of piclamilast for the treatment of asthma and COPD has been discontinued due to a particularly unfavorable oral bioavailability.

### 2.2. Quinoline Derivatives

**Figure 3:**
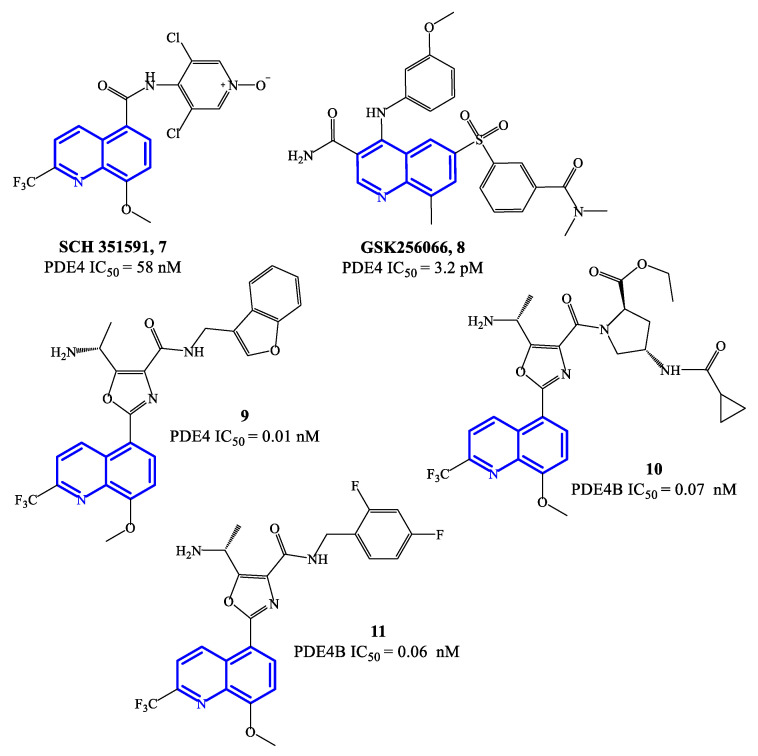
Chemical structures of PDE4 inhibitors based on a quinoline scaffold.

Selected PDE4 inhibitors based on a quinoline core are reported in Figure 3. SCH 351591 **7** (IC_50_ = 58 nM) features the 3,5-dichloropyridine moiety (present in roflumilast **2** and widely validated as a critical fragment to reach PDE4 inhibitory activity) linked at position 5 of the 8-methoxyquinoline through a carboxamide function. This agent was preclinically investigated for oral administration, providing robust efficacy data against lung inflammation [52]. SCH 365351, the metabolite of **7** (i.e., the deoxygenated product on the N-oxide of the 3,5-dichloro-4-pyridinyl residue) forming in vivo, was also a potent and highly selective PDE4 inhibitor (IC_50_ = 20 nM). Both SCH 351591 and SCH 365351 demonstarted to inhibit the production of cytokines in human blood mononuclear cell preparations [52]. Differently, GSK256066 **8** was developed for inhaled administration (mainly to treat asthma), being the inhalation route a preferential choice for the treatment of respiratory diseases. In pharmacological studies, GSK26066 was more effective than catechol ether-based PDE4 inhibitors (e.g., roflumilast **1** and cilomilast **5**), in terms of both affinity and inhibitory activity towards PDE4B [53], leading to high anti-inflammatory efficacy in clinical trials.

Compounds **9**–**11** (Figure 3) are 8-methoxyquinoline-based analogues containing a 2-trifluoromethyl group, designed as further elaboration of GSK26066. Overall the potency of these inhibitors is still in the low pM range on PDE4 [54,55]—i.e., IC_50_ = 0.01 nM for compound **9**, while **10** and **11** have IC_50_ = 0.07 and 0.06 nM, respectively. Compound **11** has also demonstrated efficacy in interfering with TNF-α release in cell-based assays. The mechanism of inhibition determined by this class of quinoline-based compounds is reported to involve the small (Q_1_) and large (Q_2_) lipophilic pockets of PDE4, by establishing interactions with the methoxy and tri-fluoromethyl groups present on the quinoline core [56], while a phenylalanine residue is involved in π–π stacking with the quinoline ring [22] and the solvent-exposed pocket (S pocket) can interact with other functional groups present in the structures.

### 2.3. Quinazolinedione and Pyrimidinedione Derivatives

**Figure 4:**
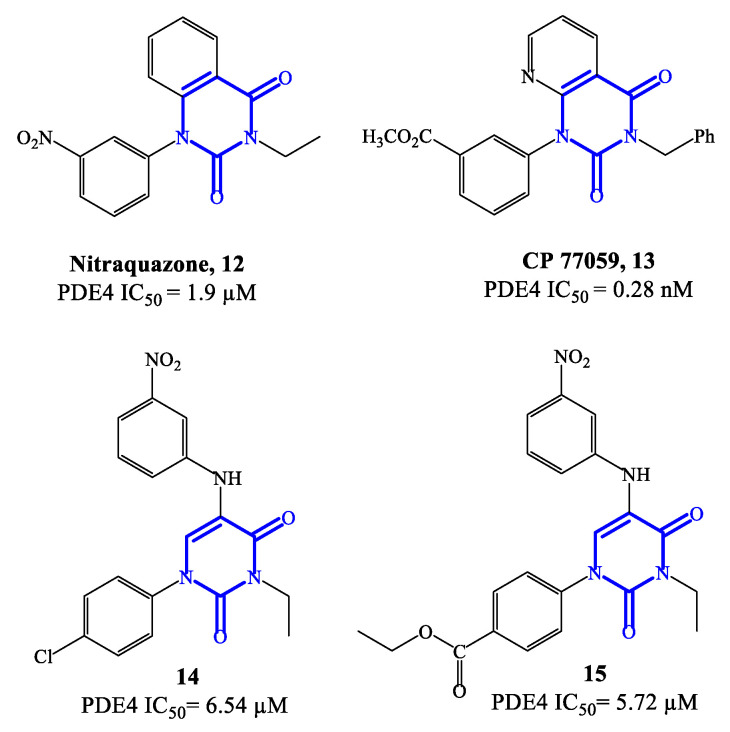
Chemical structures of PDE4 inhibitors based on quinazolinedione and pyrimidinedione scaffolds.

Nitraquazone **12** (Figure 4), the prototypical quinazolindione-based PDE4 inhibitor, was widely investigated in pharmacology studies conducted during the 1980s, to fully characterize its pronounced anti-inflammatory properties, including the capability of inhibiting the release of lysosomal enzymes during inflammation [57]. In parallel, the chemical structure of this agent was extensively manipulated in a variety of medicinal chemistry programs, leading to the development of several analogues with relevant PDE4 inhibitory activity. Structure–activity relationship (SAR) studies on the new series of derivatives highlighted that the presence of additional electron-withdrawing groups could produce benefits in terms of both affinity and activity on PDE4. Moreover, it has also been reported that the 3-NO_2_-phenyl residue in nitraquazone **12** can be replaced by an array of groups and/or functions, keeping a good level of affinity and inhibitory potency towards PDE4 [57]. CP 77059 **13** (Figure 4) is a representative example of nitraquazone derivative developed through these studies, possessing a similar planar heterocyclic structure (based on the aza-quinazolindione system), which has significant potency as PDE4 inhibitor.

Subsequently, simplified analogues of nitraquazone have also been developed, where the fused bicyclic system was replaced by a single pyrimidinedione or pyridinone core [57]. The PDE4 inhibitory potency of these series of simplified analogues is overall lower, being the IC_50_ values, in most cases, in the micromolar range. However, several 5-anilino derivatives, such as **14** (with a para-Cl-phenyl residue at position 1 of the pyrimidine-2,4-dione scaffold) and **15** (with the same structure of **14**, but with an ethyl ester that replaces the chlorine in the same position) (Figure 4) exhibited an interesting biological profile and a high selectivity for PDE4 (i.e., vs. PDE3 and PDE5). In particular, the IC_50_ values reported for **14** and **15** are 6.54 μM and 5.72 μM, respectively, and both analogues do not show affinity and inhibitory effects towards PDE3 and PDE5 [58]. This suggests that further development of new series based on these structures could lead to the identification of more potent inhibitors, also endowed with high specificity for PDE4.

### 2.4. Pyridazinone Derivatives

**Figure 5:**
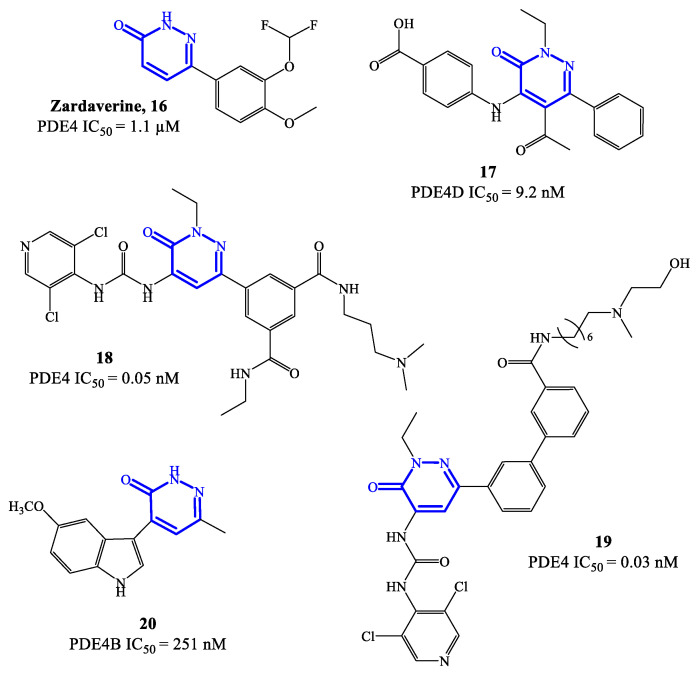
Chemical structures of PDE4 inhibitors based on a pyridazinone scaffold.

Zardaverine **16** (Figure 5) was originally reported in 1984, as a dual PDE3/PDE4 inhibitor for the treatment of asthma, demonstrating to inhibit bronchoconstriction events more effectively than theophylline [59]. Due to the very fast elimination in vivo, the clinical development of zardaverine was terminated in 1991 [27], but this pyridazinone-based molecule is still widely adopted as hit compound for the design of new analogues as PDE4 inhibitors. Through X-ray studies, it was confirmed that zardaverine forms two hydrogen bonds in the active site of PDE4D2, involving interactions between one of the catechol oxygens with Gln369 side chain (on one side of the molecule) and the pyridazinone oxygen with His160 nitrogen (on the opposite side). Compound **17** (Figure 5) was designed as further elaboration of zardaverine, through the introduction of additional substituents in positions 2, 4 and 5 of the heterocyclic ring. It was demonstrated that **17** has a higher affinity for the PDE4 active site, where the conserved Glu369 can form a hydrogen bond with the pyridazinone scaffold while the lipophilic pockets are occupied by the ethyl group in position 2 of the molecule. In following studies on this series of pyridazinone-based inhibitors, it was also established that the presence of basic residues is an essential structural requirement to reach long-term potent activity on PDE4 suitable for inhaled administration [60]. This aspect was explored with compounds **18** and **19** (Figure 5), where structural modifications of compound **17** were carried out in this regard. However, **18** and **19** demonstrated a low inhibitory efficacy in preclinical studies, as well as pro-inflammatory effects, which prevented their further development [60].

In 2021, a new series of pyridazinone derivatives as PDE4 inhibitors has been reported [61,62], showing that all the terms with an indole residue in position 4 of the heterocyclic ring possess a relatively higher inhibitory activity towards PDE4B in in vitro tests, in comparison to dihydropyridazinone analogues. This suggested that the increased planar character of the pyridazinone-4-indole derivatives translates into a better interaction in the S pockets of the active site of the enzyme, producing a more pronounced inhibitory effect. A representative example of this series is compound **20** (Figure 5), bearing a 5-methoxyindole moiety in position 4 of the pyridazinone scaffold, which was the analogue with the higher inhibitory activity within the series, associated to a good selectivity for PDE4B isoform. In in vitro tests, compound **20** was able to inhibit about 64% of PDE4B activity at 20 μM, providing an IC_50_ = 251 nM [61]. Furthermore, **20** has shown efficacy in regulating the release of potent pro-inflammatory cytokine and chemokine in cells, while no cytotoxic effects and abnormal pro-inflammatory actions were recorded. Molecular modeling studies have been carried out to elucidate the binding mode of **20** in the active site of PDE4B, highlighting the formation of hydrogen bonds (mostly by the methoxy group in position 5 of the indole moiety) with specific residues (e.g., Gln615), and van der Waals interactions (by the two aromatic systems arranged in a flat structure) with the extended hydrophobic regions. Additional in silico data indicated that a small hydrophobic cavity is located at the base of the active site, being close to the the methyl group in position 6 of the pyridazinone ring in most of the poses adopted by **20** [61]. These results might provide valid insights to access new pyridazinone-based derivatives (designed from **20**) as selective PDE4B inhibitors.

### 2.5. Pyrimidine Derivatives

**Figure 6:**
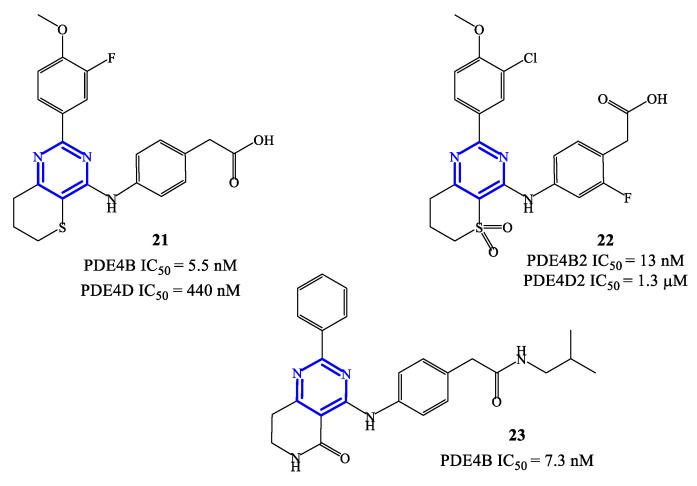
Chemical structures of PDE4 inhibitors based on a pyrimidine scaffold.

PDE4 inhibitors based on a pyrimidine core represent an elaboration of pyrimidine-2,4-dione class (see Section 2.3), which in general were endowed with moderate potency but relevant selectivity for PDE4. These features prompted molecular optimization studies based on pyrimidine as a closely related scaffold, in order to access analogues with improved potency by retaining a selective targeting towards PDE4 (and, more specifically, PDE4B), as the predominant subfamily involved in the control of inflammatory responses [58]. In this context, a series of 2-phenylpyrimidine derivatives was developed by Suzuki and coworkers [63], highlighting the analogue **21** (Figure 6) as the most promising candidate for clinical development. Compound **21** demonstrated a high inhibitory activity toward PDE4B (IC_50_ = 5.5 nM) in in vitro tests, with 80-fold lower effects recorded also on PDE4D (IC_50_ = 440 nM). The following in vivo studies also demonstrated that **21** has minimal impact in determining adverse reactions on the gastrointestinal system (e.g., by delaying gastric emptying in mice, where a 67-fold higher dose of compound **21** was needed to determine the same effects of roflumilast). However, lipopolysaccharide (LPS) inhalation-induced neutrophilia experiments indicated that compound **21** has only a relatively low efficacy in models of airway inflammation, in comparison to roflumilast [63], thus the development of new analogues was required to enable possible clinical translation.

Compounds **22** and **23** (Figure 6) were developed in this regard through chemical manipulation of the structure of **21** [63,64,65]. Compound **22** showed a relatively high inhibitory activity and selectivity toward PDE4B (IC_50_ = 13 nM on PDE4B2 and a 433-fold selectivity over PDE4D2), as well as a high therapeutic efficacy in the LPS-induced pulmonary neutrophilia in mice [64]. In parallel, the analogue **23** also showed high inhibitory activity (IC_50_ = 7.3 nM on PDE4B) and efficacy in LPS-induced TNF-α production (IC_50_ = 0.21 nM). In particular, in vivo anti-inflammatory activity tests indicated that compound **23** can inhibit 41% of LPS-induced pulmonary neutrophilia in mice when tested at a dose of 1.0 mg/kg, which is overall in line with the efficacy recorded with roflumilast used as reference in the assays [65]. X-ray studies have been conducted on compound **23** co-crystallized with a human PDE4B catalytic domain, highlighting that the pyrimidine ring forms π−π stacking interactions with Phe446 residue within the active site [64,65], while a lipophilic region is occupied by the aliphatic residue present in the structure of this inhibitor.

### 2.6. Other Fused-Heterocycle Derivatives

**Figure 7:**
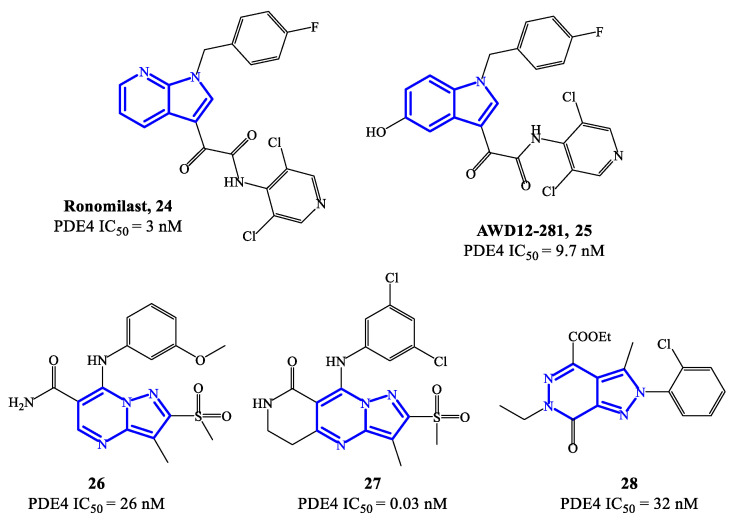
Chemical structures of PDE4 inhibitors based on fused-heterocyclic scaffolds.

Ronomilast **24** (also known as ELB353) (Figure 7) is a PDE4 inhibitor based on a 1H-pyrrolopyridine core, developed for oral administration to treat pulmonary diseases. ELB353 demonstrated an IC_50_ = 3 nM, a remarkable pharmacokinetic profile in vivo and high efficacy in inhibiting LPS-induced pulmonary neutrophilia [66]. Moreover, it showed not to trigger severe side effects in ongoing Phase I clinical trials. Compound **25** (AWD12-281) (Figure 7) is a hydroxylated indole-based derivative that was designed as an alternative for topical administration. For PDE4 inhibitors, topical route has many advantages over the oral route, such as long-term therapeutic benefits in a focused compartment of the body (e.g., the lung) and minimal systemic exposure which may limit adverse reactions. Overall, AWD12-281 was a selective PDE4 inhibitor (IC_50_ = 9.7 nM), suitable for administration via dry powder drug inhalation [67], being therefore evaluated in clinical trials for the treatment of lung inflammatory diseases, primarily COPD. Moreover, the possible use of AWD12-281 has also been investigated in acute and chronic phases of atopic dermatitis, due to the remarkable properties of penetrating human skin and determine anti-inflammatory effects.

An additional class of fused-heterocycle PDE4 inhibitors features the pyrazolo-pyrimidine as a scaffold. Among these, compounds **26** and **27** (Figure 7) are two prominent examples, possessing IC_50_ = 26 and 0.03 nM, respectively [68]. The study of these compounds has been supported by molecular modeling analysis, which was useful to explain the high inhibitory efficacy recorded in biological tests, highlighting the following interactions in the active site of PDE4B and/or PDE4D: (1) the nitrogen of the pyridine ring and the amide function interact with Gln443 via a water molecule; (2) the amide function forms also a hydrogen bond with Asn395; (3) the substituents in position 3 of the pyrazole can occupy a small hydrophobic region [68]. By varying the nature of the bicyclic scaffold, pyrazolopyridazinones were also developed as potential PDE4 inhibitors. Within this series, compound **28** (Figure 7) was the most potent, with IC_50_ values in the low nanomolar range (i.e., 32 nM). A selected docking pose adopted by this analogue into the PDE4B catalytic binding pocket shows that the ethyl ester group sits in a small binding pocket and forms two hydrogen bonds, one with the Gln443 side chain and the other with a water molecule [69]. An additional hydrogen bond is formed between the nitrogen atom in position 5 of the core structure and the Gln443 side chain, while the 2-Cl-phenyl residue can form Van der Waals interactions with other amino acid residues (i.e., His234, Ile410 and Phe414) present in the catalytic site.

### 2.7. Benzoxaborole Derivatives

**Figure 8:**
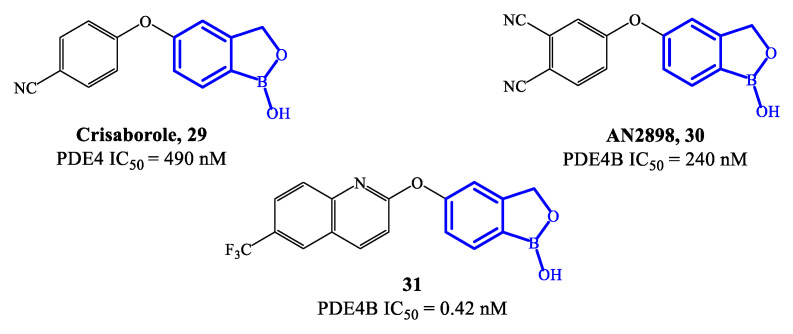
Chemical structures of PDE4 inhibitors based on a crisaborole scaffold.

Crisaborole **29** (Figure 8) is a PDE4 inhibitor which was approved in 2016 for the treatment of mild to moderate atopic dermatitis, following clinical studies confirming that it can disrupt the inflammation, inhibiting the activity of NF-ĸB and the production of inflammatory cytokines [70,71,72]. The low molecular weight makes crisaborole suitable for topical administration, with enhanced penetration of epidermis and dermis compared to other PDE4 inhibitors used into the clinics. Moreover, crisaborole is metabolized into inactive products, producing minimal systemic exposure [70]. The outcomes from two phase II clinical trials (AN2723-AD-301 and AN2728-AD-302) showed that there was a lower disease severity in patients treated with crisaborole 2% topical ointment, as well as a decrease of the lesions, associated with a favorable tolerability and safety of the drug. In comparison to other approved PDE4 inhibitors, the most common side effect of crisaborole is pain at the site of application, while gastrointestinal adverse reactions (including vomiting and nausea) have not been referred.

A new class of benzoxaborole-based analogues was reported in 2020, focusing on improving even further the therapeutic performance of crisaborole [20]. Two promising drug candidates are AN2898 **30** and compound **31** (Figure 8) [73]. In particular, **31** demonstrated outstanding inhibitory affinity (IC_50_ = 0.42 nM) and selectivity for PDE4B. In an in vivo test to establish anti-inflammatory activity (i.e., PMA-induced mouse ear oedema), **31** produced a inhibition rate of 65.85%, resulting more effective than crisaborole at the same dose [21,73]. Furthermore, the ointment containing 2% of **31** also showed higher therapeutic efficacy than crisaborole at the same concentration. In molecular modelling studies it was demonstrated that the binding of compound **31** is mainly determined by the benzoxaborole scaffold, which forms a hydrogen bond with His234 and can also establish interactions with the ion pocket in PDE4B, while the lipophilic pocket is reported to be occupied by the quinoline nucleus, which could consequently result determinant for the increased inhibitory activity [21,71]. Several in vivo toxicity studies indicated that no systemic toxicity or death was caused by a high dose of compound **31** (700 mg/kg) and no obvious differences were found in terms of physiological indexes of the animals, further supporting to continue the clinical validation of this agent.

### 2.8. Isocoumarin Derivatives

**Figure 9:**
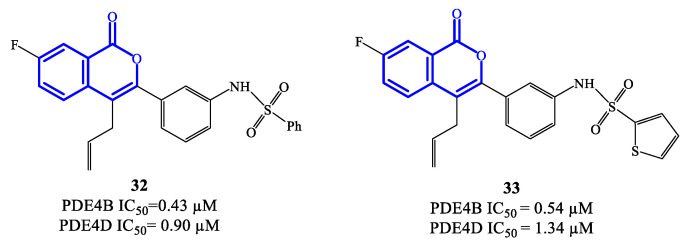
Chemical structures of PDE4 inhibitors based on a isocoumarin scaffold.

Isocoumarin-based derivatives containing in position C-3 a meta-substituted phenyl with aminocarboxamide or amino-sulfonyl groups were also developed as PDE4 inhibitors [74]. Within this series, compounds **32** and **33** (Figure 9) demonstrated to possess relatively moderate inhibitory affinity toward PDE4B, with IC_50_ = 0.43 μM and 0.54 μM, respectively. In these derivatives, the allyl group increases the lipophilic character and is considered important for improved bioavailability. Through SAR and in silico studies, it has also been confirmed that: (i) the presence of the NH (in the sulfonamide) in meta on the benzene linked to the isocoumarin at position C-3 significantly improves the inhibitory effects towards PDE4; (ii) the introduction of the sulfonamide in meta on the benzene is more effective than in para; (iii) the oxygen atoms of the sulfonamide function can form a hydrogen bond with the His450 residue in the active site of the enzyme, while the aromatic substituent can form π–π stacks with Phe586 [74]. However, the selectivity of compounds **32** and **33** towards PDE4B was limited overall, wait a IC_50_ = 0.90 and 1.34 μM on PDE4D, and further structure optimization approaches would be required to access analogues that may enable clinical evaluation [74,75].

### 2.9. Benzodioxole Derivatives

**Figure 10:**
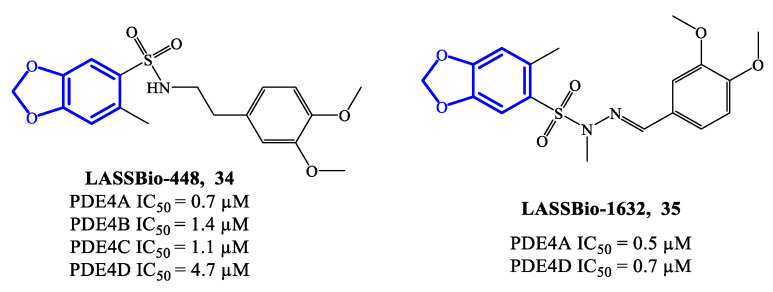
Chemical structures of PDE4 inhibitors based on a benzodioxole scaffold.

LASSBio-448 (**34**) (Figure 10) is a benzodioxole-based derivative that inhibits all four PDE4 isoforms and was originally designed as an oral PDE4 inhibitor for the treatment of asthma. LASSBio-448 has IC_50_ = 0.7 μM, 1.4 μM, 1.1 μM and 4.7 μM towards PDE4A, PDE4B, PDE4C and PDE4D, respectively [76]. It demonstrated relevant efficacy in reversing the LPS-induced lung inflammation and inhibiting several pathological conditions linked to the inflammatory state, such as mucus exacerbation, peribronchiolar fibrosis, lung eosinophilic infiltration and allergen-induced airway hyperreactivity. Moreover, in comparison to rolipram, LASSBio-448 produced lower side effects, possibly due to its moderate activity on PDE4D [76]. Molecular docking studies highlighted similarities and differences in the binding mode of LASSBio-448 towards PDE4 isoforms. Noteworthy, the 3,4-dimethoxyphenyl ring has been found to form π–π stacking interactions with Phe372 residue in all PDE4 subtypes. LASSBio-448 is also involved in van der Waals interactions with Ile336, Met337 and Phe340 in PDE4A, [76], while a hydrogen bond with the NH-group of the conserved Gln369 has been found with PDE4B. In general, the docking on PDE4B exhibited some differences in the orientation of the molecule, in comparison to the poses obtained with PDE4A. Moreover, the two oxygen atoms of the 1,3-benzodioxole ring and the sulfonyl group can form interactions with other amino acid residues in PDE4B, such as Cys358, His160 and His204. In the case of PDE4D, LASSBio-448 has also shown to form hydrogen bonds in the metal ion pocket of the active site [76,77]. In order to optimize this prototype, a novel class of derivatives containing a sulfonyl hydrazone function were synthesized and reported in 2020. Within the library, compound LASSBio-1632 (**35**) (Figure 10) inhibit PDE4A and PDE4D with IC_50_ = 0.5 μM and 0.7 μM, respectively. Moreover, compound **35** demonstrated to suppress the expression of TNF-α and inhibit LPS-induced airway hyperreactivity in lung tissues [77], opening possibilities of further preclinical validation in inflammatory disease models.

### 2.10. Furan- and Oxazole-Based Derivatives

**Figure 11:**
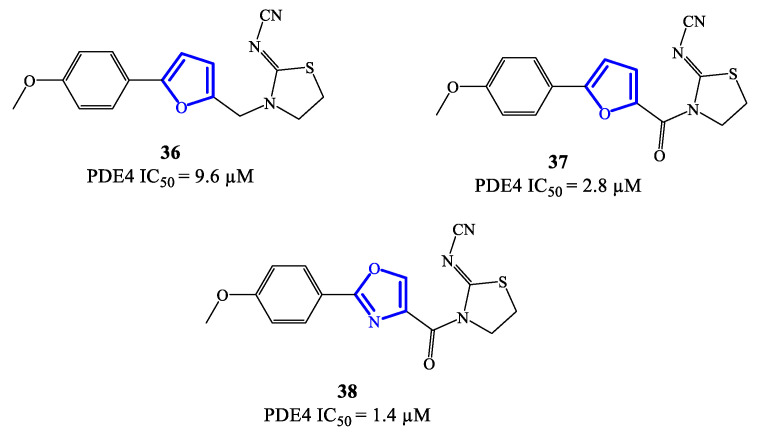
Chemical structures of furan- and oxazole-based derivatives as PDE4 inhibitors.

Five-membered heterocycles have also been investigated as scaffolds to access clinically relevant PDE4 inhibitors. In 2020, compounds **36, 37** (based on a 2,5-disubstituted furan) and **38** (based on 2,4-disubstitued oxazole) (Figure 11) were reported as PDE4 inhibitors generated through a lead optimization research program focusing on SCH 351591 **7** (i.e., a quinoline-based agent; Figure 3) [54]. The IC_50_ values recorded on PDE4 for **36**–**38** are in the micromolar range, i.e., 9.6, 2.8 and 1.4 μM, respectively [78]. Through docking studies, it has been possible to define relevant features of the binding mode of this class of inhibitors, such as: (1) the presence of an amide function linked to the heterocycle (in **37** and **38**) can strengthen the binding with PDE4 by forming a hydrogen bond with Gln443 in the hydrophobic region and, therefore, enhance the activity; (2) the thiazolidine ring contributes to the formation of hydrophobic interactions with Phe446 in the active site; (3) the oxygen of the p-methoxyphenyl group fills the ion pocket and may contribute to increase the inhibitory activity by interacting with the metal ions (i.e., Zn^2+^ and Mg^2+^) [78]. Due to their moderate activity, these new inhibitors need further development in terms of structural optimization, enabling to explore possibilities of clinical efficacy, if any.

### 2.11. Natural Products and Derivatives as PDE4 Inhibitors

**Figure 12:**
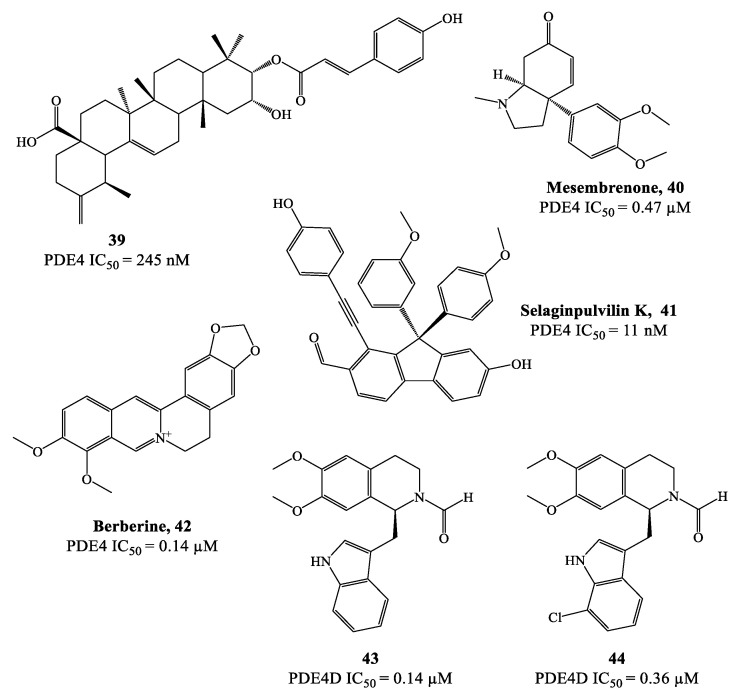
Chemical structures of natural products and derivatives as PDE4 inhibitors.

Natural products often represent attractive hits for drug development and this reflects also in the discovery of potential PDE4 inhibitors [79,80]. As a prominent example, theophylline is a nonselective PDE inhibitor which was adopted in the clinics for the treatment of COPD and asthma [79,81]. In recent years, many other natural compounds have been identified as PDE4 modulators. For instance, compound **39** (Figure 12), extracted from Gaultheria yunnanensis, was confirmed to possess moderate potency as PDE4 inhibitor (IC_50_ = 245 nM) [82], while mesembrenone **40** (Figure 12), extracted from Sceletium tortuosum, was a selective PDE4D inhibitor, with IC_50_ = 0.47 μM. Similarly, selaginpulvilin K **41** (Figure 12) is reported as a potent inhibitor of the same isoenzyme (with an IC_50_ = 11 nM), which was further supported by docking analysis, confirming that this molecule can occupy the active site of PDE4D by forming a hydrogen bond with Gln369 and π–π stacking with Phe372 [83].

**Figure 13:**
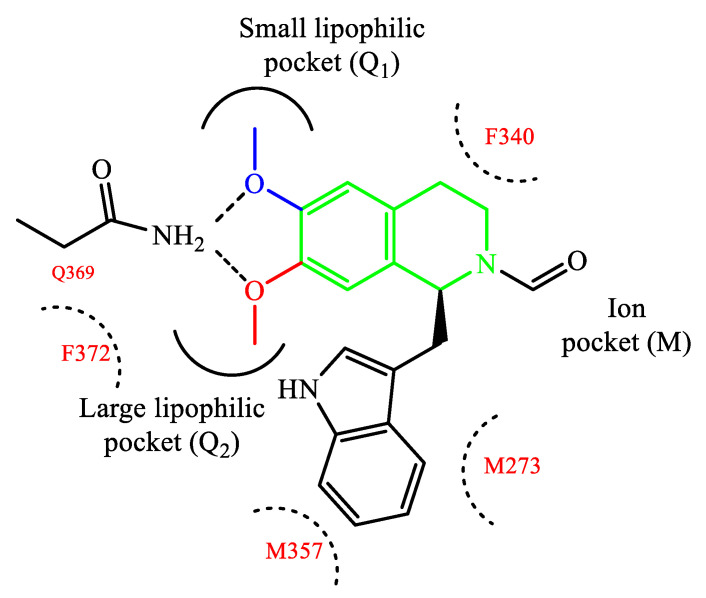
Schematic representation of compound **43** interacting with PDE4D.

Rational modification of natural product structures has also been widely explored to generate novel libraries of PDE4 inhibitors. In this regard, berberine **42** (Figure 12), extracted from Coptis chinensis, has been found to interfere with PDE4 signaling [84] (along with many other physiological functions), offering great potential for structural modifications to access new PDE4-active series. For instance, molecular simplification approaches (carried out on berberine) led to the development of novel series of tetrahydroisoquinoline-based derivatives as potential PDE4D inhibitors 85,86], including compounds **43** and **44** (Figure 12), which have been biologically investigated for the treatment of asthma, COPD and psoriasis [85]. X-ray studies showed the relevant regions of the PDE4D active site that can interact with compound **43** (for a schematic representation, see Figure 13). In particular, the small lipophilic pocket Q_1_ was entirely occupied by one of the methoxy groups, while the other is not able to fill the lipophilic pocket Q_2_ in full (being this latter larger than Q_1_). Moreover, hydrogen bonds and π−π stacking interactions have also been found in the PDE4D catalytic site with conserved residues, such as Gln369 and Phe372 [86]. Since there is no obvious interaction between the ion pocket of PDE4 and compound **43**, the development of new derivatives could focus on the introduction of functional groups which can specifically bind to the two metal ions (Mg^2+^ and Zn^2+^) in the ion pocket, as this is expected to enhance the inhibitory effects of the compounds on PDE4. With regard to compound **44** (Figure 12), this agent showed the most potent therapeutic efficacy for the treatment of psoriasis by topical administration. Although its potency (IC_50_ = 0.36 μM) is twice weaker than berberine and the analogue **43** (IC_50_ = 0.14 μM for both), as well as lower than apremilast (IC_50_ = 0.074 μM) used as reference in the biological studies [85,86], **44** demonstrated to effectively inhibit LPS-induced TNF-α production and reverse the imiquimod-induced psoriasis-like condition in murine models of skin inflammation, thus being worth of further investigation to support clinical studies.

## 3. PDE4 Inhibitors with Dual Activity

More recently, there has been increasing interest in developing drugs with dual (or multimodal) activity as the possibility of producing synergistic or additive effects, by simultaneous interference with two (or more) biological targets. This approach could result beneficial to tackle such a disease at multiple levels, positively influencing its resolution. Indeed, it is often the case that multiple signaling pathways and key proteins are usually involved in the progression of pathophysiological processes.

**Figure 14:**
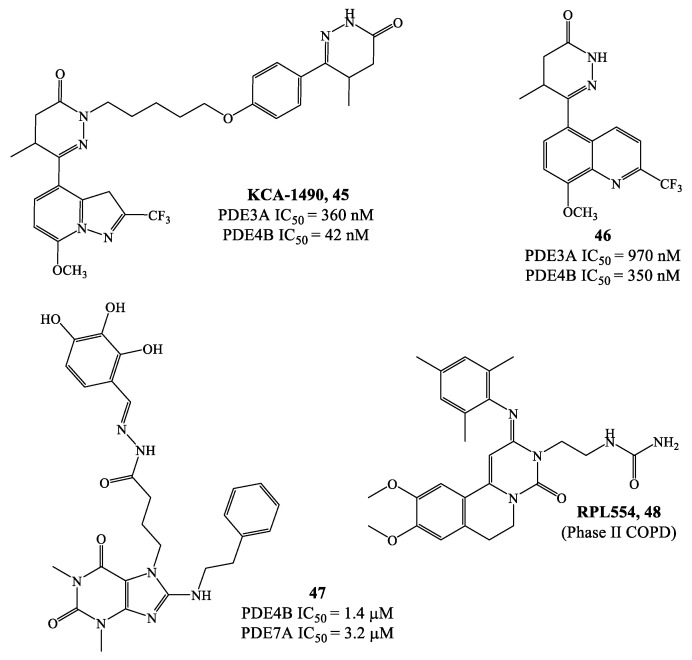
Chemical structures of PDE4 inhibitors with dual activity.

In this context, dual agents have been developed to simultaneously target PDE4 and other phosphodiesterase subtypes, or also other biological macromolecules, involved in a particular disease. For instance, dual inhibition of PDE3/PDE4 and PDE4/PDE7 has been proposed as a promising strategy to treat COPD and other inflammatory diseases to the lung. In preclinical tests, a series of dual PDE3/PDE4 inhibitors based on a dihydropyridazinone scaffold (i.e., **45** and **46** in Figure 14) and the derivatives **47** and **48** (Figure 14) have demonstrated both anti-inflammatory and bronchodilatatory properties, suggesting that they might produce advantages over the use of isoform selective inhibitors. Specifically, compounds **45** (KCA-1490) and **46** are dual agents with IC_50_ = 369/42 nM and 970/350 nM on PDE3A/PDE4B, respectively [87,88,89]. Dual PDE4/PDE7 inhibitors, such as the purine-2,6-dione butanehydrazide derivative **47** (Figure 14), were subsequently developed and showed also in this case pronounced anti-inflammatory effects due to the simultaneous inhibition of both isoforms (with IC_50_ values in the low micromolar range, i.e., 1.4 µM on PDE4 and 3.2 µM on PDE7) [90]. Further development led to the tricyclic compound RPL554 (**48**) [91,92], which is currently in phase II clinical trials for the treatment of COPD.

Dual agents simultaneously targeting PDE4 and other biological targets (e.g., as agonists/antagonists of receptors, or inhibitors of other classes of enzymes) are also well documented in the literature [93,94,95,96], including ‘PDE4 inhibitors—muscarinic receptor M3 antagonists’, ‘PDE4 inhibitors—β2 receptor agonists’, ‘PDE4 inhibitors—serotonin reuptake inhibitors’, to name a few. Overall, the rationale behind the design of these dual compounds is that joining two pharmacophores (for the two targets of interest) in a single agent may enable a more effective result towards the resolution of a disease.

**Figure 15:**
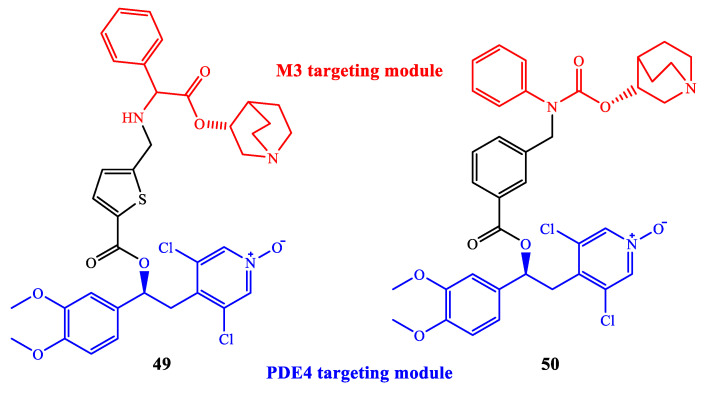
Examples of dual ‘PDE4 inhibitor-M3 antagonist’ agents.

As an example, the combination in a single agent PDE4 inhibitor and M3 antagonist action has been widely recognized as highly beneficial for the treatment of pulmonary diseases via inhaled administration. In this regard, Armani et al. recently reported a family of dual ‘PDE4 inhibitor-M3 antagonist’ molecules developed as further elaboration of CHF6001 (i.e., tanimilast, a clinically validated PDE4 inhibitor) [97,98]. The combination of an appropriately selected fragment of CHF6001 (targeting PDE4) and an established molecular system as M3 antagonist (i.e., quinuclidine nucleus), linked together in the structure through an ester or carbamate function, led to the development of a series of dual M3/PDE4 agents (e.g., see compounds **49** and **50** in Figure 15), having high potency (i.e., nanomolar) on both targets, as well as anti-inflammatory efficacy in vitro and in vivo, worth of further investigation to support clinical translation.

## 4. Conclusions

Over the past 40 years, substantial progress has been made in the discovery of PDE4 inhibitors as therapeutic agents, producing an exhaustive set of preclinical and clinical data which widely supports drug discovery programs on new treatments for inflammatory-based diseases, such as COPD, asthma and psoriasis. In this journey, many structurally different agents have been developed and pharmacologically evaluated, highlighting in some cases promising therapeutic opportunities. However, only a small number of PDE4 inhibitors are currently approved for use as drugs. This is mainly due to the adverse effects observed with several PDE4 inhibitors under development, which were initially associated to the lack of specificity in the mechanism of action and hampered the clinical development in full. In this regard, a relevant challenge concerns difficulties in generating PDE4 isoform-selective inhibitors, due to the high degree of sequence and structural similarity between the various subtypes, especially for conserved regions of the catalytic site. On the other hand, some recent studies report that the use of PDE4 isoform nonselective inhibitors can also produce high efficacy in preclinical and clinical studies, along with limited side effects. In parallel, the simultaneous inhibition of PDE4 and other PDEs (e.g., with dual PDE3/PDE4 or PDE4/PDE7 inhibitors) has highlighted synergistic effects in determining the increase of both cAMP and cGMP levels, thus favouring the resolution of diseases affected by signaling pathways involving these cyclic nucleotides. Moroever, the development of dual inhibitors simultaneously targeting PDE4 (possibly in isoform-selective fashion) and other biological targets (e.g., the muscarinic receptor M3) has also been investigated and would appear an additional direction worth of further pharmacological validation in the field.

The discovery of novel PDE4 inhibitors remains an active area of research for medicinal chemists. Our aim in preparing this review is to assist researchers with a selection of hits, along with structural requirements, which would facilitate molecular optimization strategies leading to the identification of new drug candidates. Although some agents under development possess relatively low activity/efficacy compared to approved PDE4-targeting drugs, the chemical manipulation of such prototypes still represents a valid approach to improve the overall therapeutic potential.

## Data Availability

Not applicable.
